# Demand for Crop Insurance in Developing Countries: New Evidence from India

**DOI:** 10.1111/1477-9552.12403

**Published:** 2020-09-29

**Authors:** Ranjan Kumar Ghosh, Shweta Gupta, Vartika Singh, Patrick S. Ward

**Keywords:** Agricultural risk management, crop insurance, developing countries, discrete choice experiments, India, willingness‐to‐pay

## Abstract

Determining farmers’ real demand for crop insurance is difficult, especially in developing countries, where there is a lack of formal financial sector integration and a high reliance on informal risk mitigation options. We provide some new estimates of farmers’ willingness‐to‐pay for insurance in the context of a large‐scale subsidised programme in India. We conducted a discrete choice experiment with agricultural households across four states in India, enabling us to estimate preferences for specific insurance policy attributes such as coverage period, method of loss assessment, timing of indemnity payments and the cost of insurance. Our results suggest that farmers do value crop insurance under certain conditions and some are willing to pay a premium for such coverage in excess of the subsidised rates they are currently required to pay under this programme. In particular, farmers value the assurances that they will receive timely payouts when they incur losses, and may not have a strong preference for the method with which losses are assessed. On the other hand, farmers are quite sensitive to coverage periods. Our baseline assessment shows that when optimised to farmer requirements, there can be a sizeable demand for crop insurance by developing country farmers.

## 1 Introduction

Although agricultural insurance has been extensively researched and practised, there is still not much clarity as to how much farmers value it over other methods of risk management (Skees, 2000; Jensen *et al*., 2018). Given the enormity and high exposure to multiple risks, especially in developing countries, and the problems of basis risk with index insurance (Clement *et al*., 2018), large‐scale, subsidised multi‐peril indemnity‐based crop insurance programmes have been integral to governmental strategy for risk mitigation (Hazell and Varangis, 2019). However, rarely have these programmes seen sizeable take‐up rates without explicit support through large government subsidies, and in many countries demand has been meagre even at prices well below actuarially fair rates (Yu and Sumner, 2018; Feng *et al*., 2020). Even in developed countries, there is not much evidence to suggest that risk aversion among farmers is high enough to pay for purely private actuarial premiums (Goodwin, 2001; Smith and Glauber, 2012). Traditional indemnity‐based insurance programmes are subject to a myriad of well‐documented challenges, including information asymmetries in the form of moral hazard and adverse selection (Hazell, 1992; Morduch, 2006; Barnett and Mahul, 2007; Miranda and Farrin, 2012; Wu *et al*., 2019) and ambiguity aversion (Elabed and Carter, 2015). They are also prone to other challenges, including high administrative costs (in particular, the cost of assessing losses), and covariate risks that translate into either higher risks of insolvency or which increase the cost of reinsurance. All these challenges are more pronounced in developing countries, where information asymmetries, knowledge gaps and other structural and operational issues are even more widespread. Moreover, despite a great deal of research, there remains relatively scant evidence to suggest that traditional crop insurance positively affects farmer welfare (Hazell, 1992; Smith and Watts, 2010; Fadhliani *et al*., 2019).

Coexistence of multiple problems in crop insurance markets can even lead to anomalies in farmers’ choices, for instance, in coverage periods (Babcock, 2015; Huo *et al*., 2018). India presents an interesting test case in which to explore actual demand for indemnity‐based crop insurance, as it has witnessed a significant renewed interest and an aggressive policy push since 2016. Although insurance against production loss from multiple perils has been around for many years, adoption rates have been largely disappointing. The most recent experience comes from the Pradhan Mantri Fasal Bima Yojana (PMFBY) that was launched in 2016 to replace the existing National Agriculture Insurance Scheme (NAIS).^1^ Since its launch, approximately 55 million farmers have been insured, exceeding enrolment from the previous scheme by approximately 40%, making it one of the largest crop insurance experiments in the world. Despite the low price and attractive terms, few farmers willingly purchase insurance under the new policy framework of PMFBY. Only 25% of insured farmers purchased insurance of their own volition; the remaining 75% were insured as part of compulsory loan default coverage: specifically, according to the programme’s design, any farmer who has applied for seasonal agricultural credit was mandated to purchase insurance coverage.

While there are attempts to examine farmer level risk characteristics that influence product selection, evidence is scarce on assessing valuations for individual attributes of a crop insurance. This top‐down approach – that fails to engage local stakeholders, particularly farmers, in the design process – can lead to low levels of programme acceptance among target groups and reduced chances of success for such development programmes (Feder *et al*., 1985). In the Indian context, it raises important questions about the demand for insurance and the overall viability of the existing policy. Why is voluntary participation still very low when premiums are quite affordable for almost all farmers? Is it the quality of insurance product that is a deterrent, and not the farmers’ ability to pay? What features of an insurance policy are most attractive to farmers, given their specific needs? None of these can be satisfactorily answered unless there is a fundamental assessment of farmers’ willingness‐to‐pay (WTP) for comprehensive crop insurance policies. Meanwhile, any attempt to design a large‐scale insurance policy aimed for high uptake remain not only far‐fetched, but also lack the necessary market intelligence to maximise their chances of success. We fill this gap by providing evidence on farmers’ preferences for crop insurance policy features, based on a discrete choice experiment (DCE) conducted across four geographically different states in India. The advantage of the choice experiment methodology is that it is designed not only to gauge their valuation for the totality of a crop insurance product, but also for each of the specific policy features, such as the coverage period and coverage amount (i.e., the total sum insured), the method of loss assessment and the timing of insurance payouts. Knowledge of features of a crop insurance which are more important to farmers can help policy‐makers make the necessary adjustments in existing policy and improve its overall efficiency and effectiveness. While our choice sets are agnostic to any specific insurance scheme, they include all the important attributes that are present in the current large‐scale insurance programme in India. Our results, therefore, not only contribute to the broader literature on WTP for crop insurance, especially in developing countries, but are also valuable for actual policy design in the specific Indian context.

## 2 The Problem of Insurance Demand

Determining the real demand for crop insurance has remained largely unresolved, in both developed and developing countries (Vandeveer, 2001). In an assessment of Australian wheat farmers Patrick (1988) found almost negligible willingness to pay above the actuarially fair cost, and no buyers in cases where the loading factor exceeded 20%. In another assessment in Australia, farmers were not willing to pay more than 5% of the actuarially fair cost (Bardsley *et al*., 1984). Smith and Goodwin (1996) and Smith and Goodwin (2010) have assessed crop insurance in the US and found that a typical farmer’s WTP for multi‐peril risk protection was not higher than the costs of providing the insurance. This, they argue, is not indicative of farmers’ risk aversion, but instead a reflection of farmers’ alternative risk management mechanisms such as diversification, off‐farm employment, or self‐insurance. As a result, there is hardly any crop insurance scheme anywhere in the world that is not highly subsidised. In the US and Canada, the average subsidy has been around 60% in recent times; in Spain and Portugal, subsidies have been nearly 70%; and in Japan, subsidies have been roughly 50% (Mahul and Stutley, 2010; Du *et al*., 2016).

The problem of insurance demand is further confounded in developing countries because there are many tools that governments use to smooth farm incomes, such as quotas, minimum price support systems, input subsidies and low interest agricultural loans (Mahul and Stutley, 2010). Some options promote moral hazard where a combination of high input subsidies, low interest loans and insurance lead to poor management practices in a ‘low investment – assured return’ setting (Hazell and Hess, 2010). In Burkina Faso, Sakurai and Reardon (1997) find that expectation of public food aid reduced the demand for drought insurance. Also known as a ‘Samaritan’s Dilemma’ (Coate, 1995), this is especially relevant where area‐based approaches are prevalent for both crop insurance and disaster assistance and recovery payments, such as loan waivers. In such a context, low‐risk farmers who are indemnified in an insured area may simply want to wait for a low probability disaster payment rather than investing in crop insurance. Self‐insurance (e.g., grain storage, livestock sales or leveraging social networks) is another factor that reduces demand for formal insurance in developing countries (Kazianga and Udry, 2006; Ambrus *et al*., 2014). Where insurance is compulsory (e.g., bundled together with crop loans), low‐risk farmers that have not applied for credit may be reluctant to purchase insurance knowing it cross‐subsidises high‐risk farmers (GoB, 2009). In India, a peculiar interaction further complicates the understanding of insurance demand: waivers of agricultural credit. This hampers the repayment culture and solvency of banks while at the same time not translating into higher agricultural investments or productivity (Kanz, 2016). In the Indian state of Karnataka, it has recently been reported that indebted farmers do not visit rural banks that are in charge of issuing insurance. This is out of both a fear of having to repay their loans and a hope that there will be a political intervention around an electoral event when outstanding loans would be forgiven (Ghosh, 2018).

These experiences imply that simply focusing on estimating insurance demand through observed prices (in this case, premium rates) may not yield much information. Recently there have been some limited efforts to estimate insurance demand using nonmarket valuation methods, such as the contingent valuation method (CVM) or DCEs. Liesivaara and Myyrä (2017) conducted a split sample DCE to include disaster aid as a constant variable in estimating WTP for different attributes of a crop insurance product in Finland. They found that expectations of disaster relief meant farmers would be less worried about crop losses. In such a situation, premiums would have to be highly subsidised for insurance take‐up, implying expensive use of taxpayers’ money for very low marginal benefits. Fahad and Jing (2018) use CVM to estimate the possible premium range that farmers would be willing to pay to insure themselves against risks of flooding in a high flood prone region of Pakistan. Those who said ‘yes’ to participation in an insurance product were given six starting bid levels in the range of 0.07–0.71 USD as options for the monthly premiums. There were lower and upper bounds to the dichotomous choice bids and for each choice of premium, the reasons for rejecting a higher bid were asked. This helped reveal that access to credit, irrigation, exposure to adverse weather events, and other socio‐economic constraints affected farmers’ insurance demand. Arshad *et al*. (2016) performed a double‐bound dichotomous choice CVM to elicit WTP in a hypothetical insurance market for two extreme weather events: floods and droughts. The experimental sample consisted of 240 farmers from across 12 agro‐climatic zones of Pakistan. Only 28% of respondents were willing to opt for insurance, and exhibited a very low WTP of PKR 627 (USD 4.49) per year per acre of land for drought and PKR 659 (USD 4.72) per year per acre for floods. The WTPs were inversely related to the bid values and access to canal irrigation and directly related to incomes. Although low in values, positive WTPs for crop insurance confirmed that there is a potential to develop agricultural insurance markets in Pakistan. Sherrick *et al*. (2003) used a conjoint analysis to determine the relative importance of product attributes, mainly type of indemnification (revenue vs. yield coverage), coverage election levels and their implications for frequency of payment and cost, and the insurable units available under each product (acreage flexibility). The conjoint results demonstrated the strong influence of acreage flexibility in farmers’ preferences for crop insurance. Beyond flexibility, revenue products were preferred to yield products, with the insurance type attribute, carrying roughly 30% importance, compared with 55% for flexibility.

The Liesivaara and Myyrä (2017) study evaluates attributes of an insurance product, but the relevance of the insights is largely limited to the EU context. Moreover, the focus has been on interaction with co‐risk mitigation options. The studies in Pakistan by Arshad *et al*. (2016) and Fahad and Jing (2018), on the other hand, offer meaningful insights for insurance demand in a developing country context, but have two limitations. First, the assessment is for two named perils, floods and droughts, thus limiting insights on crop insurance products. Second, they adopt a holistic CVM approach that can only speak generally of WTP for insurance. It gives no insights on how farmers value the various attributes of insurance products and thereby does not help in optimising insurance design. While Sherrick *et al*. (2003) make an attempt to address the product attribute issue, they use conjoint analysis that lacks a sound, theoretical relationship with real market choice behaviour, does not directly yield WTP and lacks a direct link with utility maximisation, or more generally random utility theory (Louviere *et al*., 2010). In this context, an assessment of WTP for crop insurance that also evaluates the preferences for attributes will help develop a more comprehensive understanding insurance demand in developing countries. This is especially true in a context where one of the largest government‐subsidised crop insurance programmes in the world (PMFBY) is currently operational as it provides an opportunity to validate outcomes. The next section describes the empirical approach adopted in the study.

## 3 Empirical Methods

We use DCEs to better understand Indian farmers’ preferences for various elements of crop insurance. Discrete choice experiments allow researchers to analyse stated preferences for products or services, but beyond that they allow researchers a means for parsing out preferences for specific characteristics or attributes of a good or service. This is particularly useful if the researcher believes, as Lancaster (1966) suggested, that it is not the good or service that is the object of utility, but rather it is from the underlying characteristics of the good or service from which utility is derived. In a DCE, preferences are elicited through survey participants’ responses to a series of hypothetical choice scenarios. For a detailed description of the random utility maximisation model to analyse stated preferences DCEs, see Appendix A in Appendix S1.

### 3.1 Experimental design

To date, there has been very little research on demand for alternative crop insurance policy specifications.^2^ In this study, we are interested in estimating farmers’ preferences for the insurance coverage period, the method of loss assessment, the delivery of insurance payments, the coverage amount (referred to in the Indian context as the ‘sum insured’), as well as their sensitivity to the cost of insurance.^3^ For the coverage period, there are several potential alternatives that insurance providers could consider. For example, under India’s PMFBY, insurance covers the entire period from pre‐sowing until after harvest. Other alternatives could include only the period from sowing until harvest, or merely the pre‐sowing or post‐harvest periods. Obviously, the longer the coverage period, the greater the exposure to crop losses, but also, consequently, the greater the cost of insuring against those loses. For loss assessment, we consider the traditional method of crop‐cutting experiments (CCEs) at the insured unit level (e.g., the *panchayat*, or collection of villages governed by a council of elders), as well as remote sensing (e.g., satellite‐based) and weather‐based indices. While CCEs may provide loss assessments that are highly correlated with a particular farmer’s in‐field experiences, they are costly to administer and highly susceptible to accidental and purposeful measurement errors. Remotely sensed‐ and weather‐based indices, on the other hand, are less expensive to administer and may eliminate problems of moral hazard, adverse selection and measurement errors, but may be less correlated with in‐field experiences (i.e., involve a basis risk). The timing of insurance payments – in particular the long delays that farmers frequently endure before receiving payouts – has been often identified as a problematic feature of crop insurance in India. It is highly relevant, therefore, to investigate whether farmers would be willing to pay a premium for an insurance policy that would provide assured, timely payments if farmers experienced a loss during the coverage period. In the DCE, we allowed for insurance policies guaranteed (i.e., with 100% certainty) to provide indemnities within 6 weeks of losses being assessed, or a more uncertain delivery schedule in which payouts may be delivered either within 6 weeks of losses being assessed (with 50% chance), or delayed for more than 6 months (also with 50% chance).

Perhaps of greatest interest to both insurance providers and their clients is the cost of insurance. In our DCE, we used three different premium rates relative to the sum insured: 2.5%, 4% and 10%. We also included in our experiment a variable capturing the insured sum of the hypothetical insurance policies. We include them not because we were especially interested in preferences for larger policies versus smaller policies (we would assume *a priori* that larger payouts would be preferable to smaller payouts), but more so because we needed a baseline against which the study participants could evaluate the policy premium and the other insurance policy characteristics. In our experiment, we allowed for the insured sum to take three possible levels, specifically INR 20,000, INR 30,000 or INR 40,000 per hectare. Table 1 summarises the various attributes of an insurance policy and their various levels included in this experiment.

**Table 1:** Attributes and attribute levels included in discrete choice experiment

Attribute	Levels			
Coverage period	Pre‐sowing to post‐harvest	Sowing to harvest	Pre‐sowing only	Post‐harvest only
Method of loss assessment	Crop‐cutting experiment at village/panchayat	Remote‐sensing (satellite) based metric	Rainfall‐based index (pays out if rainfall less than 75% of historical average)	
Timing of insurance payments	Within 6 weeks of loss assessment (100% guaranteed)	50% chance of payment within 6 weeks; 50% chance of payment more than 6 months delayed		
Insured sum	INR 20,000 per hectare	INR 30,000 per hectare	INR 40,000 per hectare	
Premium	2.5% of insured sum	4% of insured sum	10% of insured sum	

Notes

When choice sets were presented to survey participants, insured sum was converted to a monetary sum per acre of land (as opposed to per hectare), while premium was converted to a monetary amount by multiplying the premium rate by the insured sum. Choice cards were translated from English into local languages (Gujarati, Kannada or Hindi).

Because a full factorial experimental design – consisting of all possible combinations of insurance policy attributes across the competing alternatives – would be intractable in any real‐world research setting, we create a fractional factorial design that satisfied some well‐established design criteria. In our particular case, we specified an orthogonal experimental design with three generic hypothetical alternatives in each choice set, and underlying utility functions consisting of all main effects and first‐order interaction effects.^4^ The experimental design was generated using Ngene 1.1.2 (ChoiceMetrics, 2014). In sum, the experimental design resulted in a total of 36 unique choice sets, each consisting of three hypothetical alternative insurance policies. The 36 choice sets were blocked into six groups of six choice sets each. Each household in the sample was then randomly allocated to one of the six groups, and then would be expected to respond to the 6 choice sets assigned to that specific choice set group.^5^

Our primary objective is to determine farmers’ WTP for different insurance product features. Rather than estimating the choice model in preference space, we take advantage of a relatively straightforward re‐parameterisation of the utility model to permit direct estimation of WTPs (Train and Weeks, 2005; Scarpa et al., 2008; Fiebig et al., 2010; Hensher and Greene, 2011). Our basic empirical model can be written as:

(1) $$u_{\mathrm{ijt}}={\lambda_{i}\left[{\mathrm{Premium}}_{\mathrm{ijt}}\right)+\gamma }_{i1}{\mathrm{Cov}}_{2,ijt}+\gamma_{i2}{\mathrm{Cov}}_{3,ijt}+\gamma_{i3}{\mathrm{Cov}}_{4,ijt}+\gamma_{i4}{\mathrm{LA}}_{2,ijt}+\gamma_{i5}{\mathrm{LA}}_{3,ijt}+\gamma_{i6}{\mathrm{Timing}}_{\mathrm{ijt}}+\left(\gamma_{i7}{\mathrm{Sum}}_{\mathrm{ijt}}\right]+\varepsilon_{\mathrm{ijt}}$$

where Cov_2_, Cov_3_ and Cov_4_ are binary variables corresponding to insurance coverage from sowing to harvesting, coverage during pre‐sowing only, and coverage during post‐harvest only, respectively, with the coverage period extending from pre‐sowing to post‐harvest serving as the reference category. Similarly, LA_2_ and LA_3_ are binary variables corresponding to loss assessments from remote sensors and rainfall‐based indices, respectively, with loss assessments from crop‐cutting experiments at the village or panchayat level serving as the reference category. Timing is a binary variable equal to one if the insurance payment is guaranteed to be delivered within 6 weeks of the loss assessment, and zero otherwise. The Premium and Sum terms are continuous variables capturing the monetary cost farmers are required to pay insurance and the sum insured, respectively. While we previously discussed the premium as a percentage rate of the sum insured, this rate was converted into a monetary figure when participants completed choice tasks by multiplying the premium rate by the sum insured for each choice alternative. The λ term is the marginal (dis‐)utility of the insurance policy price, while the γ s are marginal WTPs for the various insurance policy attributes. In the case of the coverage period, loss assessment method and timing of payment attributes, these marginal WTPs are all with respect to what would be realised with insurance policies offered under PMFBY. For the insured sum attribute, the marginal WTP is for an additional INR increase in the maximum indemnity. We permit all marginal WTP coefficients to be randomly distributed in the population, assuming each to be normally distributed.

### 3.2 Data

The data used in the present study come from a household survey and DCE conducted with agricultural households across four Indian states (Gujarat, Himachal Pradesh, Karnataka and Uttar Pradesh). While not intended to be nationally representative, the diversity of the states covered allows for some significant heterogeneity in terms of agro‐ecological and socio‐economic conditions. The survey and DCEs were conducted from mid‐February to mid‐March 2018, with most agricultural questions targeted toward the 2017 monsoon season (approximately late June–November, 2017, depending on location). Data were collected with the assistance of Agricultural Economic Research Centres (AERCs) of the Ministry of Agriculture in India. Three representatives from each of the AERCs based out of the four states and independently recruited survey enumeration staff were trained for data collection using the SurveyCTO computer‐assisted personal interviewing (CAPI) platform on Android tablets. From each of the four states, two districts were sampled based on their share of primary crop cultivation in the state. Within each district, two blocks were randomly selected, with three villages subsequently selected at random from each block. Within each village, 12 households were randomly selected from village lists, resulting in an initial sample of 576 households. Respondents were administered a set of survey questions that sought information on their demographic characteristics, cultivation practices, household asset ownership, income sources and experience with insurance policies, translated in their local languages. The DCEs were also administered using standardised, scripted protocols (see Appendix B in Appendix S1). Both the survey questions and the DCE were administered in the primary language in each state (Gujarati in Gujarat, Kannada in Karnataka, and Hindi in both Uttar Pradesh and Himachal Pradesh). Table 2 summarises the characteristics of households in the sample on both a pooled and state‐wise basis.

**Table 2:** Descriptive statistics of sample households

	Full sample	Gujarat	Himachal Pradesh	Karnataka	Uttar Pradesh
Age (years)	48	52	48	44	48
	(0.54)	(1.02)	(1.08)	(1.05)	(1.10)
Gender (proportion male)	0.86	1.00	0.59	0.89	0.97
	(0.01)	–	(0.04)	(0.03)	(0.01)
Farming experience	24.55	26.09	25.86	21.39	24.84
	(0.57)	(1.16)	(1.19)	(1.02)	(1.11)
General caste (proportion)‘	0.50	0.70	0.74	0.30	0.24
	(0.02)	(0.04)	(0.04)	(0.04)	(0.04)
‘Other backward class (proportion)	0.34	0.20	0.04	0.42	0.68
	(0.02)	(0.03)	(0.02)	(0.04)	(0.04)
Scheduled tribe/Scheduled caste (SC/ST; proportion)	0.16	0.10	0.22	0.25	0.08
	(0.02)	(0.03)	(0.03)	(0.04)	(0.02)
Area cultivated during monsoon season 2017 (acres)	5.39	9.61	2.25	6.43	3.32
	(0.31)	(0.71)	(0.29)	(0.85)	(0.24)
Total grain harvested during monsoon season 2017 (kg)	4988	8825	1434	6218	3545
	(532)	(1522)	(497)	(1307)	(326)
Primary monsoon season crop is rice (proportion)	0.70	0.94	0.01	0.85	1.00
	(0.02)	(0.02)	(0.01)	(0.03)	–
Primary monsoon season crop is maize (proportion)	0.22	0.00	0.85	0.01	0.00
	(0.02)	(0.00)	(0.03)	(0.01)	–
Duration of primary crop from sowing to harvest (months)	3.99	4.12	3.53	4.75	3.56
	(0.04)	(0.06)	(0.07)	(0.09)	(0.05)
Insured during monsoon season 2017 (proportion)	0.11	0.11	0.06	0.14	0.14
	(0.01)	(0.03)	(0.02)	(0.03)	(0.03)
Insured during monsoon season 2017 because accessed credit (proportion)	0.09	0.11	0.06	0.08	0.12
	(0.01)	(0.03)	(0.02)	(0.02)	(0.03)
Number of observations	572	142	144	142	144

Note

Standard errors in parentheses.

## 4 Results

### 4.1 WTP estimation

Table 3 presents results of our base estimates of WTP for product features under two specifications: column (1) reports results from a generalised multinomial logit regression assuming that WTP parameters are uncorrelated, while column (2) reports results permitting free correlation of the WTP parameters. Under both specifications, we permit both preference and scale heterogeneity, with WTPs for all policy attributes assumed to be normally distributed. The upper panel in Table 3 reports the estimates of the mean WTP for the corresponding attributes, while the lower panel reports the corresponding distribution parameters (standard deviations). Although there is not a sizeable difference in model fit between the two specifications (likelihood ratio test *P*‐value of 0.4), nor are there dramatic changes in the WTP coefficient estimates, the model permitting free correlation in WTP parameters is preferred to the more restrictive specification assuming that the WTP parameters are uncorrelated. When we permit correlations, all of the means and standard deviations for the WTPs are statistically significant, indicating that not only are the mean WTPs significantly different from zero, but also that there is a significant amount of heterogeneity in insurance policy preferences within the population. Indeed, when we allow for correlated WTP parameters, the standard deviations of the WTP distributions are all greater than when we preclude correlations. For the discussion that follows, we will rely upon the estimates assuming the WTP parameters are correlated. Figure A1 (Appendix S1) illustrates the empirical distributions of farmers’ conditional mean WTPs for the various insurance policy attributes considered in the present study.

**Table 3:** Willingness‐to‐pay from the discrete choice experiment: Generalised multinomial logit regression estimates

	(1)	(2)
	Uncorrelated	Correlated
Willingness‐to‐pay estimates		
Coverage level: sowing to harvest	−1.03***	−0.97***
	(0.19)	(0.21)
Coverage level: pre‐sowing	−5.18***	−4.99***
	(0.51)	(0.50)
Coverage level: post‐harvest	−4.92***	−4.74***
	(0.44)	(0.43)
Loss assessment: remote sensing	−0.36*	−0.37*
	(0.20)	(0.21)
Loss assessment: rainfall index	−0.22	−0.34*
	(0.19)	(0.21)
Timing: guaranteed within 6 weeks	1.05***	1.05***
	(0.16)	(0.18)
Sum insured (Rs. 1,000)	0.11***	0.11***
	(0.01)	(0.01)
Distributions of willingness‐to‐pay		
SD (Coverage level: sowing to harvest)	1.40***	2.26**
	(0.32)	(0.40)
SD (Coverage level: pre‐sowing)	2.88***	3.65***
	(0.41)	(0.43)
SD (Coverage level: post‐harvest)	1.71***	3.31***
	(0.42)	(0.41)
SD (Loss assessment: remote sensing)	1.83***	1.91***
	(0.29)	(0.31)
SD (Loss assessment: rainfall index)	1.86***	2.17***
	(0.32)	(0.30)
SD (Timing: guaranteed within 6 weeks)	0.34	0.91**
	(0.36)	(0.29)
SD (Sum insured (Rs. 1,000))	0.04*	0.04**
	(0.02)	(0.02)
SD (Scale parameter)	1.10***	1.03***
	(0.16)	(0.20)
Number of choice observations	3,450	3,450
Number of choice sets per individual	6	6
Number of individuals	575	575
Log‐likelihood function value	−3,092.50	−3,070.60
Number of iterations	71	184
Number of Halton draws used in simulation	1,000	1,000

Notes

Standard errors in parentheses. Models estimated in WTP‐space (Train and Weeks, 2005) using the ‘gmnl’ package (Sarrias and Daziano, 2017) in R v. 3.6.3. Estimates in column (1) assume that WTP estimates are independent, while estimates in column (2) permit free correlation in WTP estimates. All WTPs are assumed to be normally distributed in the population.

*** Significant at 1% level;

** Significant at 5% level

* Significant at 10% level.

For insurance policy attributes that enter the utility function as binary variables (coverage level, loss assessment and timing of indemnity payments), the omitted category is always the insurance policy feature that exists in policies issued under PMFBY. Consequently, many of the estimated WTPs reported in Table 3 can be interpreted either as premia that individuals would be willing to pay (in the case of guaranteed indemnity payments within 6 weeks of loss assessment) or discounts that would be demanded (in the case of coverage period or loss assessment methods) for modifications to the insurance policies that farmers can currently procure under PMFBY. For example, farmers in our sample would require discounts for policies with shorter coverage periods (i.e., anything other than pre‐sowing to post‐harvest). These required discounts are quite sizeable for policies that cover only the pre‐sowing period (INR 5,000) or only the post‐harvest period (INR 4,800). Both of these required discounts exceed the INR 3,000 that we might expect farmers to be willing to pay for a hypothetical base policy with an INR 30,000 insured sum. Other things equal, therefore, we would not expect farmers to purchase any policy that covers only these tail ends of the agricultural season. There is a smaller (though still non‐trivial) discount requirement for policies covering cropping from sowing to harvest (INR 1,000). In sum, although it seems farmers would not be interested in policies that protect against risks either leading up to or following the monsoon season, they also clearly perceive some risk of crop loss due to sources apart from just rainfall (whether deficiencies or excesses) which presumably would be covered by a policy covering the sowing to harvest period.

Farmers would demand a discount for insurance that assessed losses by remote sensing or rainfall indices, though the discount that would be demanded is not huge (roughly INR 400 for each of the two alternative methods). Farmers evidently prefer to have losses assessed by CCEs conducted at the insured unit level, despite the fact that the costs of CCEs make the insurance policies more expensive for the insured.^6^ Farmers would be willing to pay substantially more for insurance if they could believe that payments would be timely. On average, farmers would be willing to pay a premium of over INR 1,000 if indemnity payments would be guaranteed within 6 weeks of the loss assessments.

Interestingly, the estimates suggest that, on average, farmers would be willing to pay more than the heavily subsidised premiums they are currently paying under PMFBY. The WTP coefficient associated with the sum insured is approximately 0.1, meaning that if the sum insured rises by one unit (here, 1 unit equals INR 1,000), they would be willing to pay roughly INR 100, or 10% of the sum insured, though there is considerable heterogeneity around this mean. While this figure is close to the actuarially fair cost of insurance (14%), it is still quite low relative to what would likely be needed for insurance to be commercially viable without large government subsidies. For example, for a base policy with an insured sum of INR 30,000 per hectare, these results suggest that on average farmers would be willing to pay up to INR 3,000 per hectare. Under PMFBY, where farmers are typically only asked to pay at most a premium of 2% of the sum insured, the cost to farmers is only INR 600.

What does this WTP imply in terms of the potential benefits that insurance could provide to the insured? Since the sum insured represents the maximum indemnity that could be paid by the insurance contract, these results might be interpreted as implying that farmers would be willing to pay for insurance that would compensate them at 10 times the rate that they are paying. If farmers were convinced that losses were highly likely, this might appear quite reasonable, since that would entail a fairly gratifying return on their insurance premium payments. But if this were indeed how farmers were thinking about this risk transferral opportunity, it would be rather simplistic and short‐sighted, since farmers would only receive the full insured sum as an indemnity under the very rare instance of a total crop failure.

To get a better sense of the potential benefit of insurance for the farmers in our sample, the premium should be interpreted in light of the probability that they would incur a loss, and the claim that farmers would be entitled to in such a case. PMFBY has only been around since 2015, so there are not sufficient data to directly observe how frequently these policies pay out. In order to provide this more nuanced lens, we rely on historical data on district‐level yields obtained from the Indian Department of Economics and Statistics (DES) from 1998 through 2018. Using the criteria for determining claims under PMFBY (see footnote 3), we are able to roughly approximate the potential claims that farmers would have been entitled to under PMFBY had the policy been in place as far back as 2004.^7^ On average, based on the historical frequency with which actual yields fell below the threshold yields (themselves based on the parameterisation used under PMFBY), farmers in our sample could expect to suffer an insurable loss about 7.3% of the time, though this varies from state to state. In Gujarat, for example, rice yields between 2004 and 2018 never fell below the threshold yield to trigger an indemnity. In Raichur district of Karnataka, on the other hand, actual rice yields fell below the threshold yield nearly 29% of the time between years 2004 and 2018. When there is an indemnifiable loss, it is on the order of about INR 1,500. This would suggest that farmers in our sample would be willing to pay premia of roughly INR 1,000 for a potential payout of INR 1,500, but they would only expect to realise this payout <10% of the time, yielding an expected annual payout of less than INR 150.

From the perspective of private insurers, this would appear quite favourable, as it would indicate a collection of premiums that exceeds claims. This would be a particularly favourable development in the Indian context, where farmers’ claims have exceeded premiums collected under a number of previous policy regimes. Mahul *et al*. (2012) note that, under the National Agricultural Insurance Scheme (NAIS), from 2000 to 2008 the producer loss ratio (the ratio of claims paid to premiums received) was 3.5, meaning insurers were paying out more than three times what they were collecting in premiums.^8^ This is clearly unsustainable and is one of the reasons that the Indian government has sought to reform the structure of its crop insurance programmes. The results above imply a loss ratio of considerably less than the 0.95 loss ratio that is generally regarded as a break‐even loss ratio for commercial insurance (Miranda, 1991). But this runs somewhat contrary to what is frequently observed on the demand side, namely that farmers are unwilling to purchase crop insurance if the expected benefits (i.e., the expected payouts) are less than twice the premiums paid. Part of this could be explained by the fact that the mean WTP masks a considerable amount of underlying heterogeneity, as clearly illustrated in the empirical distribution of farmers’ WTP in Figure A1 (Appendix S1; bottom left panel). This distribution is bimodal, with the most prominent mode suggesting a significant portion of famers would only be willing to pay around 2.5% of the sum insured, on par with the premium rate that farmers are required to pay for crop insurance under PMFBY. The second mode is less prominent but indicates that there are some farmers in the sample willing to pay closer to 12.5% of the sum insured for coverage.

A second potential explanation could be the hypothetical nature of the DCE. Despite efforts at ensuring that respondents took the experiment seriously, there is the potential that the valuations that were estimated through these stated preferences could be upwardly biased, since there is no financial cost for the choices that are made. A recent meta‐analysis (Penn and Hu, 2018) reviewed 280 stated preference studies (e.g., DCEs and contingent valuation studies) and found the Calibration Factor (the ratio of hypothetical WTP to real WTP) to be nearly 2, suggesting that people state that they are willing to pay roughly twice what they would actually pay in a real market environment. While we are not able to determine such a Calibration Factor in our study, if we rely on this meta‐analytic result to inform our interpretation, we might reasonably conclude that actual WTP might be closer to 5% of the sum insured, implying that farmers in our sample might be willing to pay a premia of roughly INR 500 for a potential payout of INR 1,500, though, again, they might only expect to incur a loss once every 10 years.

As previously mentioned, our preferred estimates in column (2) of Table 3 allow for free correlation of the WTP estimates. Table A4 (Appendix S1) reports the covariance, correlation and Cholesky (lower) decomposition matrices that reflect the relationships among these WTP estimates. In particular, the correlation matrix provides details into how preferences co‐move. For example, preferences for certain and timely indemnity payments are negatively correlated with preferences for all the other product features, except loss assessments via remote sensing. This correlation is not especially strong (0.147), but it is nonetheless interesting that increasing valuations for guaranteed, timely indemnity payments are positively correlated with valuations for insurance policy designs that increase the likelihood of timely payment delivery.

In addition to controlling for correlated preferences, we also considered the salience of the different attributes when respondents are evaluating choice scenarios. To evaluate this, we consider inferred attribute non‐attendance using the method proposed by Hess and Hensher (2010). We use the individual‐level estimates of the WTP distributions for the various insurance policy attributes and compare the uncertainty in individual WTP estimates relative to the expected WTP level. Specifically, we compute a noise‐to‐signal ratio by dividing the standard deviation of the WTP distribution by the absolute value of the mean WTP for each individual and for each policy attribute. These noise‐to‐signal ratios are then compared to a threshold value that is used to infer that respondents were not attending to specific product attributes when responding to the choice scenarios.^9^ Table 4 reports the proportion of respondents in the sample who were deemed to have not attended to the different insurance policy attributes based on this procedure. In most applications where such attribute non‐attendance is considered, non‐attendance is typically assumed to imply that an individuals’ WTP for a specific attribute would be zero. In our case, because the majority of the insurance policy features that are considered are valued relative to the features in prevalent insurance policies in India, if someone is deemed to not attend to a particular attribute, we interpret that to imply that they would neither pay a premium to have that particular feature, nor would they require a discount relative to the prevalent crop insurance policies under PMFBY.

**Table 4:** Non‐attendance to insurance policy attributes

Insurance policy attribute	Proportion not attending to attribute
Coverage level: sowing to harvest	0.19
Coverage level: pre‐sowing	0.10
Coverage level: post‐harvest	0.05
Loss assessment: remote sensing	0.68
Loss assessment: rainfall index	0.54
Timing: guaranteed within 6 weeks	0.29
Sum insured (Rs. 1,000)	—

Note:

Respondents are deemed to have ignored the insurance policy attribute if the noise‐to‐signal ratio from the respondents’ WTP distribution is >2 (Hess and Hensher, 2010).

With the exception of the loss assessment methods, most of the insurance policy features that we consider appear to be quite strongly attended to. No one appears to have ignored the sum insured – obviously an important feature of any insurance policy and the one most intimately related to the cost of insurance. Somewhat surprising, about 30% of respondents appear to have ignored the timing of indemnity payments, though delays in processing payments is often cited as a weakness of crop insurance under PMFBY. There are more nuanced results when it comes to the coverage period. Nearly 20% of respondents appear to have ignored the coverage period if it covered the sowing to harvest period, but a smaller set of respondents appears to have ignored the coverage period if it was either only the pre‐sowing period (10%) or only the post‐harvest period (5%). For those that ignore the sowing to harvest coverage period, we can infer that they essentially view the policy as *indistinguishable* from one that covered the full pre‐sowing to post‐harvest period (like those policies offered under PMFBY) and would therefore not demand a discount on the purchase of such a policy relative to a typical PFMBY‐type insurance policy. A similar phenomenon arises for the alternative methods of loss assessment. More than 50% of respondents ignored each of the two alternative loss assessment methods. One might suspect that these respondents are just not paying attention to the method with which losses are assessed, but this is not entirely accurate. There is a very weak correlation (0.17) between respondents’ behaviour when it comes to attending to or ignoring these two loss assessment methods. Roughly 80% of the farmers in our sample view loss assessments from crop‐cutting exercises as being virtually indistinguishable from other methods of loss assessment, though these farmers do not necessarily feel equally ambivalent among remote sensing or weather‐based indexing methods.

### 4.2 Determinants of WTP

Using the individual‐level conditional estimates of WTP, we next aim to isolate any systematic correlates of farmers’ WTP for the different insurance policy attributes. In so doing, we assume that attribute non‐attendance (as defined above) indicates that the farmer would neither be willing to pay a premium nor demand a discount for the policy feature vis‐à‐vis the PMFBY‐type policy. Table 5 presents two sets of estimates. First, in columns (1)–(7), we present OLS regressions of the individual‐level mean WTPs for the various insurance product attributes as a function of various individual‐ and farm‐level characteristics. Each column pertains to the regression for the WTP of the different insurance policy attributes. In these regressions, we implicitly assume that the attribute‐specific WTPs are independent, though we note that the WTP estimates are derived from the generalised multinomial logit regression that allows for free correlation of the WTPs. In column (8), we present random effects regressions that allow for the WTPs for the different attributes to be correlated within individuals (e.g., Campbell, 2007; Yao *et al*., 2014). By and large, the results do not suggest much in the way of systematic determinants of WTP for the insurance policy features considered in the present study, perhaps confirming the old adage *de gustibus non est disputandum*.^10^ There are some interesting and statistically significant effects that emerge. We find that farmers who primarily cultivate rice during the monsoon season have a significantly higher WTP for insurance policies with loss assessments based on remote sensing (vis‐à‐vis farmers who primarily cultivate non‐cereals during the monsoon season). This is a promising result, given that there have already been researchers and development practitioners working on the ground in India and other countries (largely in south‐eastern Asia) piloting remote sensing for rice yield prediction to eventually inform crop insurance programmes.^11^ The predictive accuracy of the remote sensing technologies has been rather encouraging, with predictive accuracy ranging between 85% and 96% across three sites in Tamil Nadu (a state at the southern tip of India) when predictions were made at the block (sub‐district administrative unit) level, compared with accuracy of 87% when predictions were made at the district level (Pazhanivelan *et al*., 2015). The high spatial resolution and high – and increasing – predictive accuracy of these remote sensing technologies is a promising development, and the pre‐eminence of rice cultivation across much of India would suggest a nascent market for such products.

**Table 5:** Determinants of willingness‐to‐pay for insurance contract attributes

	OLS							RE
Dependent variable:	(1)	(2)	(3)	(4)	(5)	(6)	(7)	(8)
WTP for:	Coverage period: sowing to harvest	Coverage period: pre‐sowing	Coverage period: post‐harvest	Loss assessment: remote sensing	Loss assessment: rainfall index	Timing: guaranteed within 6 weeks	Sum insured (INR 1,000)	WTP (INR 1,000)
Intercept	−1.43***	−5.14***	−4.90***	−0.71	0.47	1.12***	0.08***	−0.40
	(0.83)	(1.95)	(1.78)	(0.60)	(0.79)	(0.36)	(0.03)	(0.63)
Gender (male = 1)	0.40**	0.50	0.62*	0.06	0.19	−0.06	−0.01	0.25*
	(0.17)	(0.40)	(0.37)	(0.12)	(0.16)	(0.07)	(0.01)	(0.13)
Age (years)	0.03	0.01	0.004	0.002	−0.04	−0.01	0.79	−0.001
	(0.04)	(0.09)	(0.08)	(0.03)	(0.03)	(0.02)	(0.001)	(0.03)
Age^2^ (×1,000)	−0.06	0.25	0.24	−0.01	0.45	0.01	−0.01	0.12
	(0.36)	(0.85)	(0.78)	(0.26)	(0.34)	(0.16)	(0.01)	(0.27)
Experience (years)	−0.03*	−0.01	−0.02	−0.01	−0.01	0.01	−0.52	−0.010
	(0.02)	(0.05)	(0.04)	(0.01)	(0.02)	(0.01)	(0.68)	(0.02)
Experience^2^ (×1,000)	0.22	−0.50	−0.31	0.15	0.18	0.02	0.02	−0.03
	(0.33)	(0.78)	(0.72)	(0.24)	(0.32)	(0.14)	(0.01)	(0.25)
Area cultivated (acres)	0.002	0.03	0.01	−0.01	0.004	−0.002	−0.002	0.01
	(0.02)	(0.04)	(0.04)	(0.01)	(0.02)	(0.01)	(0.61)	(0.01)
Area cultivated^2^ (×1,000)	−0.04	−0.001	−0.68	0.21	−0.27	0.13	0.01	−0.26
	(0.38)	(0.90)	(0.82)	(0.28)	(0.36)	(0.17)	(0.01)	(0.29)
Primary crop: rice	0.13	0.02	0.20	0.66***	0.21	−0.02	−0.83	0.17
	(0.24)	(0.57)	(0.52)	(0.18)	(0.23)	(0.11)	(0.01)	(0.18)
Primary crop: maize	−0.24	−0.90	−0.72	0.28	−0.45*	0.09	0.01	−0.28
	(0.28)	(0.66)	(0.60)	(0.20)	(0.27)	(0.12)	(0.01)	(0.21)
Crop duration (days)	−0.01	0.06	0.04	−0.04	−0.04	−0.02	−0.001	−0.002
	(0.06)	(0.14)	(0.13)	(0.04)	(0.06)	(0.03)	(0.002)	(0.05)
Insured (=1)	−0.17	0.50	0.35	−0.05	−0.02	−0.02	−0.01	0.08
	(0.16)	(0.38)	(0.35)	(0.12)	(0.16)	(0.07)	(0.01)	(0.12)
Caste: OBC	−0.28**	−0.36	−0.31	−0.01	−0.03	0.01	0.002	−0.14
	(0.13)	(0.32)	(0.29)	(0.10)	(0.13)	(0.06)	(0.01)	(0.10)
Caste: SC/ST	0.01	0.38	0.32	0.07	0.18	−0.07	−0.01	0.13
	(0.15)	(0.36)	(0.33)	(0.11)	(0.15)	(0.07)	(0.01)	(0.12)
Coverage period: sowing to harvest								−0.72***
								(0.09)
Coverage period: pre‐sowing								−3.57***
								(0.09)
Coverage period: post‐harvest								−3.40***
								(0.09)
Loss assessment: remote sensing								−0.32***
								(0.09)
Loss assessment: rainfall index								−0.32***
								(0.09)
Timing: guaranteed within 6 weeks								0.63***
								(0.09)
*R* ^2^	0.03	0.08	0.07	0.02	0.01	0.04	0.04	0.52
No. observations	571	571	571	571	571	571	571	571

Notes

Standard errors in parentheses. Each regression controls for state‐level fixed effects. Dependent variable in each regression is the conditional mean (marginal) WTP for each of the insurance policy characteristics estimated by the generalised multinomial logit regression (see Table 3), adjusted for inferred attribute non‐attendance (see Table 4).

*** Significant at 1% level;

** Significant at 5% level;

* Significant at 10% level.

We also found that farmers who primarily cultivate maize during the monsoon season have a significantly lower WTP for insurance policies with loss assessments based on weather indices (vis‐à‐vis farmers who primarily cultivate non‐cereals during the monsoon season). Interestingly, 369 out of the total 372 farmers who primarily cultivate maize during the monsoon season are from Himachal Pradesh, a state with very large climatic variations due to differences in altitude. These large variations in climate conditions may increase the likelihood that realised weather conditions on a farmer’s field may not match the realised weather conditions at the location where the weather data comprising the index are collected, thus increasing the basis risk for insured farmers, which could be one of the primary reasons why maize farmers may dislike index‐based loss assessments. Finally, we find evidence of a U‐shaped relationship between cultivated area and farmers’ WTP for crop insurance policies with loss assessments based on remote sensing technologies. Initially, WTP for crop insurance based on remote sensing is declining with increasing cultivated area but begins to increase after farm sizes exceed 43 acres.^12^

## 5 Discussion

Our results suggest that, other things equal, farmers would generally be interested in purchasing crop insurance similar to the products being offered under PMFBY, and furthermore at premium rates higher than they are currently being asked to pay. If that is indeed the case, then it is puzzling that crop insurance coverage remains so low in India and seems to be declining. Recent reports suggest that gross cropped area covered by insurance policies under PMFBY fell by more than 20%, 59.55 million hectares in 2016–17 to 47.5 million hectares in 2017–18.^13^ This remains less than 24% of gross cropped area in the country. At the same time, the number of insured farmers has also decreased by 14%, from 55 million to about 48 million. Related to the sluggish – and declining – enrolments, one of the major challenges that policy‐makers in India face regards the long time delay in delivering indemnity payments. There is also anecdotal evidence that insurance company representatives – who take part in the crop cutting exercises (CCEs) – lower the threshold limit below which indemnities are issued, so that even farmers with substantial crop losses may not qualify for payment.

Even before these recent declines, only about one third of farmers in India were insured. One obvious reason for the lack of coverage is that many farmers simply do not know about this programme. From our data, nearly 35% of farmers across these four states had never heard of PMFBY. Furthermore, because crop insurance is typically compulsory for farmers accessing loans, there is evidence that insurance companies do not consider non‐loanee farmers to be profitable (Ghosh, 2018). It is also possible that transaction costs in acquiring insurance and filing claims could hinder the uptake. For example, farmers may have to travel several kilometres to reach the nearest financial institution. From our data, roughly 10% of farmers indicated that they would not likely purchase insurance if they had to travel far to submit the requisite paperwork for acquiring insurance. Moreover, certain requirements such as land records exclude tenant farmers from participating in PMFBY.

How can this ambitious government policy be amended to increase coverage rates and better meet the needs of Indian farmers? Clearly, the most pressing need is to expedite the delivery of indemnity payments. But it seems unrealistic to expect this to be implemented without concomitant changes to the way in which agricultural losses are assessed. The time associated with undertaking the requisite number of CCEs, not to mention the sometimes dubious manner in which these experiments are conducted, not only diminishes the external validity of the yield estimates but also makes it incredibly difficult to process and distribute accurate indemnity payments.^14^ Assessing crop losses via other means, such as remote sensing technologies, provides a means for more rapidly assessing crop damages, which in turn should facilitate more timely processing of indemnity payments. And given that nearly 80% of the farmers in our sample seem indifferent between loss assessments via CCEs and those from other methods, transitioning to these alternative methods would seem a viable candidate for improving the structure and design of crop insurance policies to best suit farmers’ demands. However, some caution is also necessary, since there is a problem with basis risk associated with, especially, index‐based products, and also with remote sensing data. There is a trade‐off between prompt payments (generally requiring less individually specific assessment of losses) and basis risk which we have not been able to explore in our experiment.

**Figure 1:**
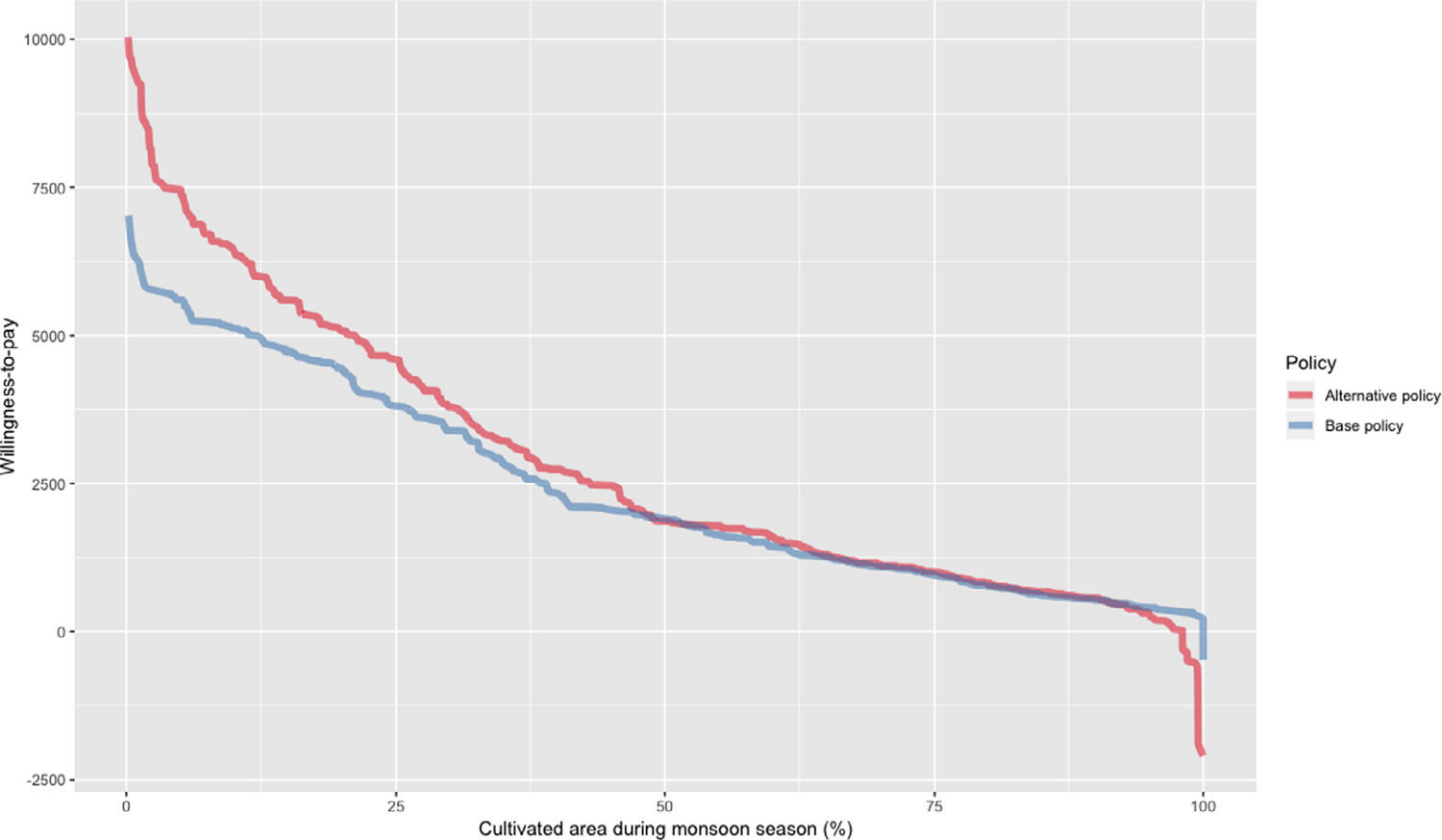
Demand curves for competing crop insurance packages: Base policy similar to those offered under PMFBY vs. alternative policy offering more timely delivery of indemnity payments under remote sensing

How would insurance demand and farmer welfare change as the result of such a transition? Figure 1 plots empirical demand curves for two crop insurance products: a base policy similar to those being offered under PMFBY, and an alternative policy that has been modified so that losses are assessed via remote sensing, but also one that offers a guarantee that indemnity payments will be delivered within 6 weeks of the losses being detected. The horizontal axis depicts the percentage of cultivated area covered (or not) under crop insurance. Consequently, at any point at which the demand curve for the alternative policy is above the demand curve for the base policy, we would expect a higher proportion of cultivated area to be insured at a given price. For virtually all prices above INR 1,800 per hectare (representative of a 6% premium on an insured sum of INR 30,000 per hectare), demand for the alternative policy exceeds demand for the base policy. For example, at a price of INR 2,100 per hectare (representative of a 7% premium on an insured sum of INR 30,000 per hectare), we would expect roughly 46% of cultivated area to be insured under the alternative policy, but only 43% of cultivated area to be insured under the base policy. This is not a large margin, but achieving 46% coverage of cultivated area is a marked improvement on the existing achievements under PMFBY. Consequently, farmer welfare would also be enhanced by these alternative products, with the increasing surplus derived from two sources. First, as just mentioned, there would be an increase in the cultivated area that would be covered under this type of alternative crop insurance product, which presumably would translate into a larger number of insured farmers. Second, some farmers would derive greater value under this alternative type of policy than they would under a PMFBY‐type policy.

## 6 Conclusion

In developing country production systems, insurance is only one of the many tools governments use to smooth farm incomes such as quotas, minimum price support systems, input subsidies and low interest crop loans. There are interactions with other risk management programmes which make it difficult to understand the actual valuation for an insurance product based on pure observed data or revealed preferences. With a push for large‐scale crop insurance schemes such as the PMFBY in India, a fundamental requirement for their sustainability is to evaluate local farmers’ own preferences for various features of an insurance product including the cost. In this context, lacking any substantial information in the existing literature, we try to assess farmers’ preferences for various crop insurance features. There are estimated to be around 198 million farmers under some form of crop insurance in the developing world, especially India, China, Pakistan and Nepal (Hess *et al*., 2016; Fahad and Jing, 2018; Budhathoki *et al*., 2019). The direct and indirect cost of subsidies for agricultural insurance globally is substantial, with some fair estimations putting the figure close to $20 billion per year (Hazell and Varangis, 2020). Yet, the coverage is quite low and voluntary participation is not high. Our study shows that, over and above affordability and post‐premium subsidisation, it is the design and operation of the crop insurance instruments that limit uptake. We focus on the attributes of the insurance product which might be optimised for greater attractiveness and uptake in similar developing contexts.

We employed a DCE with a sample of Indian farmers and directly estimate farmers’ WTP for various insurance product attributes allowing for preference and scale heterogeneity and without imposing unrealistic restrictions on farmers’ sensitivity to insurance premiums. This approach has two main advantages. First, DCEs are based on the random utility theory and hence preference representation is their inherent feature. The distributions for WTP that our estimates yield support this. Second, since we show how farmers value different features of an insurance policy, our results indicate how the existing crop insurance programme can be improved by incorporating these preferences. Our results suggest that farmers are generally willing to pay higher than the subsidised premiums they are currently paying under PMFBY. While farmers prefer loss assessments through crop‐cutting experiments, they also have a very strong preference for assurances that indemnities will be paid in a timely fashion (e.g., within 6 weeks of the loss assessment).

When we consider the transition from the PMFBY‐type of crop insurance policy to an alternative policy that incorporates assessment of losses via remote sensing with guaranteed payment of indemnities in a timely fashion, we find that there are significant welfare gains to farmers, especially if we consider the lower cost of insurance that could result from eliminating the need for crop‐cutting experiments. While we are not able to directly estimate the magnitude of these welfare effects on a national basis, we can easily qualify or characterise these impacts. First, even when we abstract from cost considerations, the excess value that is associated with the alternative policy vis‐à‐vis the base policy structure (i.e., total difference in area under the demand curves) is significant. When there is a change in the cost of insurance (which we assume because of the reduction in insurers’ costs due to eliminating the need for crop‐cutting experiments), there are two effects: the increase in cropped area (which presumably translates into a more‐or‐less proportional increase in the number of insured farmers) and the increased surplus experienced by farmers that would already be insured now paying a lower price. Finally, to the extent that the higher WTP and lower cost of this alternative policy structure reduces the need for government subsidies, there is a reduction in the marginal excess tax burden needed to finance these subsidies.

This study points to several avenues for future research. To start with, researchers or policy‐makers may wish to expand upon the choice experiment design used in the present study to consider other insurance product attributes. The attributes considered in the present study were thought to be the most salient in farmers’ decision making, but any choice experiment design requires simplification and subjectivity. In addition, because there is no financial consequence of choices made in our experiment, there is the potential for hypothetical bias to inflate the estimated WTP relative to what farmers actually would pay if they were to engage in real insurance markets. This is a common criticism of choice experiments, though some authors have argued that the ability of such stated preference data to engender the estimation and prediction of real market behaviour is comparable to those of revealed preference data. Nevertheless, future research needs to consider valuation elicitation methods that might be less prone to hypothetical bias. Piloting insurance programmes with alternative crop insurance designs would provide more concrete insight into the potential for modified insurance products to increase the number of insured farmers and the total cultivated area insured. The implications of this kind of work are enormous, given the scarce formal risk management methods available to farmers in the developing countries. If optimally designed crop insurance programmes are made available and implementation bottlenecks are removed, then, as our results suggest, large‐scale crop insurance can be useful in alleviating agricultural development and food security problems.

## Supporting information


**Appendix S1.** Supplemental materials.

## Footnotes

^1^ ‘Pradhan Mantri Fasal Bima Yojana’ is translated as ‘Prime Minister’s Crop Insurance Scheme,’ but for our purposes we will simply refer to it as India’s new crop insurance programme or PMFBY. For more details, refer to Ghosh (2018).

^2^ Ye *et al*. (2017) and Wang *et al*. (2019) are notable exceptions of studies using similar methods to address demand for crop insurance in China. Using a labelled choice experiment design, Wang *et al*. (2019) evaluate farmer demand for traditional yield insurance, as well as price, revenue and weather index insurance, which are currently at the experimental stage in China. Ye *et al*. (2017) use a generic choice experiment to evaluate preferences for crop insurance attributes such as liability, franchise (percentage of yield loss rate), inspection and payment method, subsidy rate, and premium rate, and conduct policy simulations to estimate the welfare effects of alternative liability and subsidy rate combinations.

^3^ In the Indian context, the sum insured is meant to be equivalent to the scale of finance for cultivating a given crop on a per hectare basis (declared and notified by the State‐Level Coordination Committee on Crop Insurance, SLCCI), adjusted by the actual area cultivated by a specific farmer. It should be noted that the sum insured is meant to reflect the scale of farmers’ finance, as determined by the maximum indemnity that could be paid, and would only equal the actual claim amount in the case of a total crop failure. Participants were advised of this in the course of scripted instructions that were read to the participants prior to commencing the choice experiment, and that, for any case in which yields were greater than zero, the claim amount would be adjusted proportionally. The actual amount of the claim would be equal to $\mathrm{Claim}=\frac{\mathrm{Threshold}\mathrm{Yield}‐\mathrm{Actual}\mathrm{Yield}}{\mathrm{Threshold}\mathrm{Yield}}\times \mathrm{Sum}\mathrm{Insured}$ where the ‘threshold yield’ is determined as ThresholdYield=AverageYield×Indemnitylevel and the ‘average yield’ is the average yield from the preceding 7 years at the district level (excluding up to 2 ‘calamity years’), and the indemnity level takes values of 70%, 80% or 90%, pertaining to high risk, moderate risk and low risk areas, respectively. These indemnity levels would vary by district, by season and by crop.

^4^ Orthogonal experimental designs have the properties of attribute balance and independent estimability of all parameters. In practice, this implies that each attribute column in the design matrix is uncorrelated.

^5^ Since crop insurance take‐up rates are still quite low in India, there was a concern that providing respondents with an opportunity to opt‐out or to revert to their status quo would result in a high enough proportion of opt‐out or status quo that we may not be able to learn much about the potential for improving upon existing insurance programme structures. Consequently, the choice experiment did not include a status quo or opt‐out option. While this design choice has implications for the estimated WTPs, since it forces respondents to make a choice, Veldwijk *et al*. (2014) has demonstrated a remarkably high level of consistency in choices with vs. without opt‐out options, and though WTP estimates differ between datasets with vs. without an opt‐out option, there is no systematic variation. If we assume that the inclusion of an opt‐out option would have affected the estimated WTP for each of the attributes proportionally, the estimated WTPs reported in the present study may be viewed as upper bounds on the true WTP for crop insurance among Indian farmers.

^6^ While it is difficult to estimate the cost of conducting a CCE, and the costs will likely vary widely from state to state, we can roughly estimate the cost based on historical data. For example, we know that conducting a CCE cost about INR 300 in 2004 (World Bank, 2007). Based on general inflation levels over the ensuing 14 years, this corresponds to a 2018 equivalent price of about INR 500 per CCE. This assumes that, for example, wages for the agricultural workers responsible for the crop cuts just keep up with the cost of living. For remotely sensed or index‐based loss assessments, the variable costs are essentially nil. Consequently, the administrative loads on these types of policies would be considerably less, thereby lowering the cost of insurance – something that would clearly be attractive for farmers.

^7^ One factor in determining claims is the threshold yield, which is a percentage (based on a crop‐ and district‐wise indemnity level) of the average yield from the preceding 7 years, excluding up to 2 ‘calamity years’. Even though the data from DES goes back to 1998, there is not a 7‐year average that can be used for determining the threshold yield until 2004. There is no strict guidance for states in classifying calamity years, so we assume that a calamity year is any year in which average yields are either at least 30% lower than the previous year or at least 30% lower than the average yield from the preceding 5 years. The indemnity levels that are used in these calculations are based on state level insurance company notifications from the most recent year available for the survey districts in our study.

^8^ The loss ratio has improved under PMFBY, reaching 0.75 in Kharif 2018 (Financial Express, 2019).

^9^ Following Hess and Hensher (2010), we rely on a threshold noise‐to‐signal ratio of 2 to indicate attribute non‐attendance.

^10^ This Latin phrase literally translates as ‘In matters of tastes, there can be no dispute’, but is often paraphrased as ‘There is no accounting for tastes’.

^11^ A prominent example is the Remote sensing‐based Information and Insurance for Crops in Emerging Economies (RIICE) project, funded by the German Development Corporation (GIZ) and the Swiss Agency for Development and Cooperation, and led by the International Rice Research Institute (IRRI) in collaboration with Sarmap and SwissRe.

^12^ There are a couple of caveats though. First, the linear area effect is not statistically significant at conventional levels, so there may not be the initial negative relationship between area and WTP. Second, very few households in our sample – and, indeed throughout much of India – cultivate areas in excess of 43 acres. Consequently, the observed relationship may be a mere statistical aberration that should not likely have any bearing on actual agricultural policy.

^13^ See, for example, Business.

^14^ Ghosh (2018) estimates that between 1,500,000 and 2,000,000 crop cutting exercises are required each season. The report finds that these crop cutting experiments are ‘highly ill‐managed’ and ‘prone to human error and manipulation’.
