# Supplementary material for: Demand for Crop Insurance in Developing Countries: New Evidence from India
Source: J Agric Econ. 2020 Sep 29;72(1):293–320. doi: 10.1111/1477-9552.12403 (PMC7821195; doi:10.1111/1477-9552.12403)
Supplement: Supplementary file 1 — Appendix S1. Supplemental materials. [file JAGE-72-293-s001.docx]

**Demand for crop insurance in developing countries: New evidence from India**

Ranjan Kumar Ghosh, Shweta Gupta, Vartika Singh, and Patrick S. Ward

**On-line Appendix A**

# A.1. *Random utility model*

We assume that observed choices arise from a process of utility maximization (McFadden, 1974). Specifically, within the context of a discrete choice experiment, it is assumed that the observed (stated) choice that an individual makes within a particular choice scenario is the choice that, on average, maximizes her utility among the set of potential alternatives. Utility can be conceived of as consisting of both a systematic, deterministic component, and a stochastic component. The deterministic component reflects individual tastes and preferences that map the expression of product characteristics directly into utility, while the stochastic component reflects, among other things, random variations in tastes and preferences and errors in optimization. We can write our random utility model as:

| $u_{ijt}={\alpha_{i}p_{ijt}+x}_{ijt}^{'}\beta_{i}+\varepsilon_{ijt}$ | (1) |
| --- | --- |

where $u_{ijt}$ is the observed indirect utility (i.e., the utility of the utility maximizing option $j$) obtained by individual $i$ during choice scenario $t$; $p_{ijt}$ is the price of option $j$ faced by individual $i$ during choice scenario $t$; $x_{ijt}$ is a vector of (non-price) insurance policy characteristics or attributes; $\alpha_{i}$ represents individual $i$’s preferences for policy price; $\beta_{i}$ is a vector of preference weights for the corresponding elements of $x_{ijt}$; and $\varepsilon_{ijt}$ is a Gumbel (Extreme Value Type I) distributed error term with farmer-specific variance ${Var(\varepsilon}_{ijt})=\sigma_{i}^{2}(\pi^{2}/6)$, where $\sigma_{i}$ is a farmer-specific scale parameter. In many applications, it is assumed that there is no heterogeneity in this scale parameter, and furthermore the scale parameter is simply normalized to 1 for ease of computation (i.e., $\sigma_{i}=\sigma=1$). Taking partial derivatives of $u_{ijt}$ with respect to the attributes provides estimates for the change in utility associated with incremental changes in the expression of the attributes; in other words, the $\beta_{i}$ terms can be directly interpreted as marginal utilities. The ratio of two marginal utilities is directly interpretable as the marginal rate of substitution between the two attributes (i.e., the rate at which an individual would be willing to give up a unit of the attribute in the denominator to acquire an increment of the attribute in the numerator). If one of the marginal utilities is the marginal utility of income, then the marginal rate of substitution with respect to income is an estimate of WTP. We are rarely able to directly observe the marginal utility, but this can be proxied by the marginal disutility of product cost. Since cost is almost always deemed to be one of the important features driving purchase decisions, it is almost universally included as an attribute in a DCE. An estimate for the WTP for a specific attribute would therefore just be the ratio of the marginal utility of the attribute to the marginal disutility of product price.

If one assumes that, in addition to homogeneity in the scale parameter, preferences are fixed in the population, then estimating marginal utilities and arriving at estimates for WTP is relatively straightforward using conditional logit estimation. The assumption of fixed (or constant) preferences in the population is quite restrictive, however, and imposes some potentially unrealistic assumptions on, among other things, the substitution patterns that are permitted by the model. A common approach to incorporating preference heterogeneity is to estimate the choice model using a mixed logit (also known as a random parameter logit) model. Under this approach, the researcher assumes a distribution for the preference parameters, and derives an estimate for WTP as the ratio of the random parameters. This approach, however, can lead to distributions for WTP that have undefined moments (e.g., the ratio of two normally distributed random variables takes a Cauchy distribution, for which neither the mean nor the variance are defined).

# A.2. *Estimation in willingness-to-pay space*

Even if we permit preference heterogeneity, there is still the potential violation of scale homogeneity. If we permit scale heterogeneity, then we cannot simply proceed with a conventional mixed logit estimator. Note that, since utility is ordinal, we can divide equation (1) by the scale parameter to obtain a scale-free equivalent (Scarpa et al., 2008):

| $u_{ijt}=\left( \alpha_{i}/\sigma_{i} \right)p_{ijt}+x_{ijt}^{'}\left( \beta_{i}/\sigma_{i} \right)+\nu_{ijt}$ | (2) |
| --- | --- |

where, now, $\nu_{ijt}$ is an i.i.d error term with constant variance ${\pi^{2}}/6$. We can re-write the re-scaled utility coefficients as

| $u_{ijt}=\lambda_{i}p_{ijt}+x_{ijt}^{'}\psi_{i}+\nu_{ijt}$ | (3) |
| --- | --- |

Importantly, note that if $\sigma_{i}$ varies randomly in (2), the utility coefficients in (3) will be correlated, since $\sigma_{i}$ enters into the denominator of each of the re-scaled utility coefficients. Even if $\sigma_{i}$ does not vary, the utility coefficients could still be correlated simply due to correlations among tastes for various attributes (Scarpa et al, 2008). Since the WTP for a given attribute is the ratio of the marginal utility of that attribute to the marginal (dis-)utility of the policy price, we can write $\gamma_{i}=\psi_{i}/\lambda_{i}$, and can re-write (3) as

| $u_{ijt}=\lambda_{i}\left[ p_{ijt}+x_{ijt}^{'}\gamma_{i} \right]+\nu_{ijt}$ | (4) |
| --- | --- |

which re-parameterizes utility in WTP space rather than preference space (Train & Weeks, 2005; Scarpa et al., 2008). Now, rather than assuming the distributions for the marginal utilities (the $\beta_{i}$ terms), the researcher can directly specify the distribution for (individually-scaled) WTP (the $\gamma_{i}$ terms) without having to worry about ratio distributions with undesirable properties. Consequently, researchers have much more direct control over the distributional features of marginal WTP in the underlying population under this specification than they would otherwise (Thiene & Scarpa, 2009). Furthermore, Train & Weeks (2005) and Hensher and Greene (2011) have found that this transformed model generally produces more reasonable estimates of WTP than when WTP is calculated as the ratio of utility parameters. This model can then be estimated by appealing to the generalized multinomial logit (GMNL) model developed by Fiebig et al. (2010), which Hensher and Greene (2010) have demonstrated is a generalization of choice models estimated in both preference space as well as WTP space.

To allow for even greater flexibility in estimation, we consider the possibility that the randomly distributed WTPs for the different insurance product attributes could be correlated. As was previously mentioned, if there is scale heterogeneity, then WTPs will be correlated by definition, and even if there is no scale heterogeneity, there is the possibility for correlated WTPs simply due to correlation among preferences for different attributes. Hensher, Rose, and Greene (2015) have further noted that, in virtually all data sets, there are likely unobserved effects that are correlated among alternatives in a given choice situation and allowing for WTP parameters to be correlated is one way to account for this. Failing to control for this can lead to imprecise estimates of WTP, which has obvious implications for the reliability of the policy implications that be derived from these estimates (Mariel & Meyerhoff, 2018).

Expanding equation (4) based on the discussions in Section 3.1 (main text) of the product attributes, our base utility function accounting only for main effects can be written as:

| $u_{ijt}={\lambda_{i}\left[ {Premium}_{ijt} \right.+\gamma}_{i1}{Cov}_{2,ijt}+\gamma_{i2}{Cov}_{3,ijt}+\gamma_{i3}{Cov}_{4,ijt}+\gamma_{i4}{LA}_{2,ijt}+\gamma_{i5}{LA}_{3,ijt}+\gamma_{i6}{Timing}_{ijt}+\left. \gamma_{i7}{Sum}_{ijt} \right]+\varepsilon_{ijt}$ | (5) |
| --- | --- |

where ${Cov}_{2}$, ${Cov}_{3}$, and ${Cov}_{4}$ are binary variables corresponding to insurance coverage from sowing to planting, coverage during pre-sowing, and coverage during post-harvest, respectively, with the coverage period extending from pre-sowing to post-harvest serving as the reference category. Similarly, ${LA}_{2}$ and ${LA}_{3}$ are binary variables corresponding to loss assessments from remote sensors and rainfall-based indices, respectively, with loss assessments from crop-cutting experiments at the village or panchayat level serving as the reference category. $Timing$ is a binary variable equal to one if the insurance payment is guaranteed to be delivered within six weeks of the loss assessment, and zero otherwise. The $Premium$ and $Sum$ terms are continuous variables capturing the monetary cost farmers are required to pay insurance and the insured sum, respectively. While the premium attribute was previously discussed as a percentage rate of the insured sum, this rate was converted into a monetary figure when participants completed choice tasks by multiplying the premium rate by the insured sum for each choice alternative.


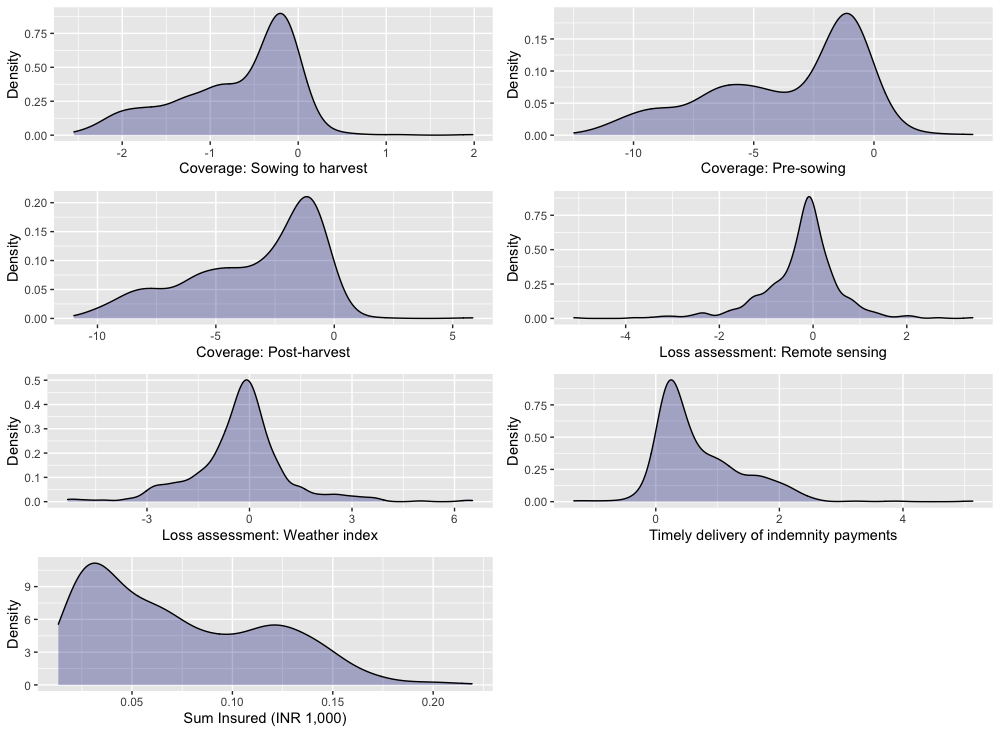


Figure A1. Empirical distributions of WTP for various insurance policy attributes

Table A4. Covariance, correlation, and Cholesky decomposition matrices from generalized multinomial logit estimation permitting free correlation in WTP parameters

| Covariance matrix | | | | | | | |
| --- | --- | --- | --- | --- | --- | --- | --- |
|  | Cov_2_ | Cov_3_ | Cov_4_ | Loss_2_ | Loss_3_ | Timing | Sum |
| Cov_2_ | 5.127 | 3.762 | 5.096 | 0.925 | 1.518 | -0.322 | -0.006 |
| Cov_3_ | 3.762 | 13.340 | 9.493 | -2.385 | 2.113 | -1.279 | -0.018 |
| Cov_4_ | 5.096 | 9.493 | 10.967 | -0.222 | 3.169 | -0.704 | -0.079 |
| Loss_2_ | 0.925 | -2.385 | -0.222 | 3.651 | 1.160 | 0.301 | -0.009 |
| Loss_3_ | 1.518 | 2.113 | 3.169 | 1.160 | 4.717 | -0.360 | -0.041 |
| Timing | -0.322 | -1.279 | -0.704 | 0.301 | -0.360 | 0.837 | -0.008 |
| Sum | -0.006 | -0.018 | -0.079 | -0.009 | -0.041 | -0.008 | 0.002 |
| Correlation matrix | | | | | | | |
|  | Cov_2_ | Cov_3_ | Cov_4_ | Loss_2_ | Loss_3_ | Timing | Sum |
| Cov_2_ | 1 | 0.455 | 0.680 | 0.214 | 0.309 | -0.156 | -0.060 |
| Cov_3_ | 0.455 | 1 | 0.785 | -0.342 | 0.266 | -0.383 | -0.121 |
| Cov_4_ | 0.680 | 0.785 | 1 | -0.035 | 0.441 | -0.232 | -0.575 |
| Loss_2_ | 0.214 | -0.342 | -0.035 | 1 | 0.280 | 0.172 | -0.120 |
| Loss_3_ | 0.309 | 0.266 | 0.441 | 0.280 | 1 | -0.181 | -0.461 |
| Timing | -0.156 | -0.383 | -0.232 | 0.172 | -0.181 | 1 | -0.203 |
| Sum | -0.060 | -0.121 | -0.575 | -0.120 | -0.461 | -0.203 | 1 |
| Cholesky (Lower) Decomposition Matrix | | | | | | | |
|  | Cov_2_ | Cov_3_ | Cov_4_ | Loss_2_ | Loss_3_ | Timing | Sum |
| Cov_2_ | 2.264 | 0 | 0 | 0 | 0 | 0 | 0 |
| Cov_3_ | 1.662 | 3.252 | 0 | 0 | 0 | 0 | 0 |
| Cov_4_ | 2.250 | 1.769 | 1.665 | 0 | 0 | 0 | 0 |
| Loss_2_ | 0.408 | -0.942 | 0.316 | 1.580 | 0 | 0 | 0 |
| Loss_3_ | 0.670 | 0.307 | 0.671 | 0.610 | 1.831 | 0 | 0 |
| Timing | -0.142 | -0.321 | 0.110 | 0.014 | -0.136 | 0.826 | 0 |
| Sum | -0.002 | -0.004 | -0.039 | 0.000 | -0.007 | -0.007 | 0.006 |

**On-line Appendix B**

**Instructions:** Please read through these instructions slowly and carefully. After the instructions are completed, you will begin going through the actual choice experiment. For this, you will need to use the set of choice cards for the group to which this household has been allocated, as provided in your tablet. Do not in any way attempt to guide the respondent in their choices. You may help the respondent to understand the differences between the hypothetical alternatives (options A and B and C), but do not attempt to suggest that one option is better than the other based on these differences. Although participants will not be asked to actually purchase the insurance products they choose in this exercise, you should nonetheless, encourage the respondent to provide choices that accurately reflect the choice he or she would make in the ‘real world.’

In this exercise, you will be asked to make choices about different types of crop insurance schemes. Please note that these are not any form of presently ongoing schemes, but an attempt to determine the characteristics of an effective crop insurance scheme that is preferred by the consumers i.e., farmers.

There are six (6) such choice scenarios that you will face. In each scenario, you will have three options, Option A, Option B, and Option C. Each of the three options describe an alternative hypothetical crop insurance product. Each choice scenario should be thought of as a menu of available policies, of which you should select your preferred policy. It is important that your choices in this exercise most accurately reflect choices you would make in the real world when faced with these scenarios. We ask that you take your time and think carefully about your options before making your selection.

While evaluating the options, you should consider only the characteristics listed, and assume that the options are the same in all other ways. For example, all the options reflect the same crop (for example, paddy), same season (for example, Kharif 2018) and for your land.

You should treat each of the six (6) choice scenarios independently. In other words, in the second choice scenario, do not think back to the options that were available in the first choice scenario. Do not make or adjust your decision based on characteristics found in previous choice scenarios.

The features that characterize the different insurance policies are the coverage period, the method by which losses are determined (and hence whether claims are to be distributed), the timing of when payouts are distributed, the sum insured, and the premium that the farmer pays. Each of these features will have several levels. We will now discuss each of these features and how they are represented across the different insurance options.

1. ***Coverage Period***: This feature refers to the period during which your crop is covered by the insurance policy. Four possible scenarios have been included:

| 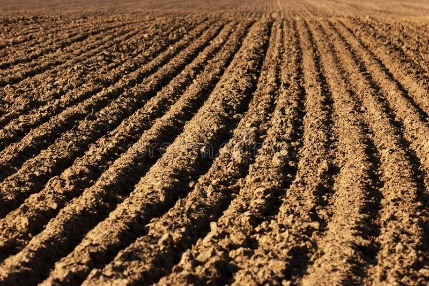 | **Pre-planting stage:** This type of policy only pertains to crop losses that are incurred due to some adverse conditions or event that occurs before planting, such as deficit rainfall or other seasonal conditions that prevent or delay sowing or transplanting. If the policy covers only the pre-planting stage, it provides no protection for losses that are incurred due to events that occur after the crop has been sown or transplanted. |
| --- | --- |
| 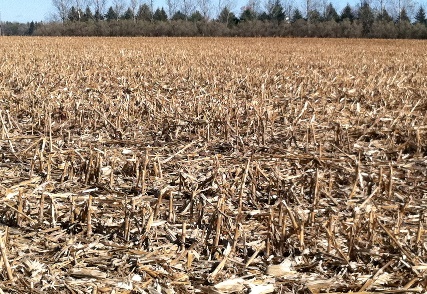 | **Post-harvest:** This type of policy only pertains to crop losses that are incurred due to some adverse condition up to two weeks after the crop has been harvested, such as due to cyclones or unseasonal rains. If the policy covers only the post-harvest stage, it provides no protection for losses that are incurred due to events that occur before the crop has been harvested. |
| 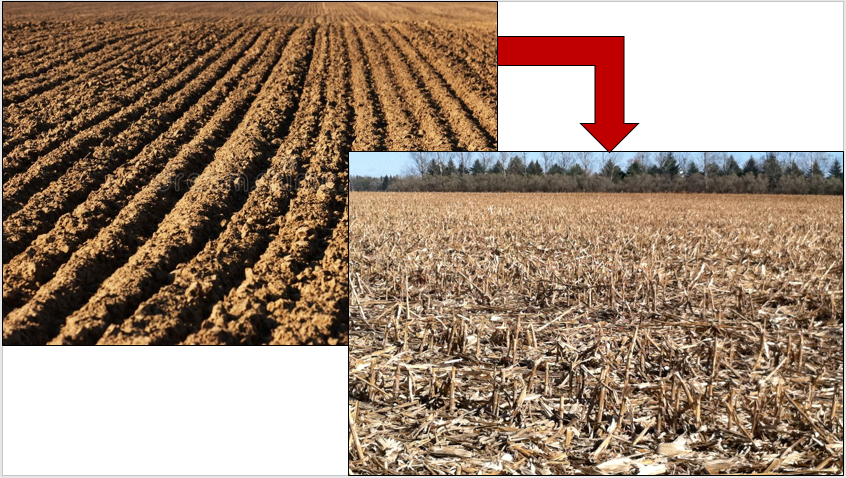 | **Pre-planting to post-harvest:** This type of policy pertains to crop losses that are incurred from before seeds are sown until up to two weeks after the crop has been harvested. This is the most comprehensive coverage period. This type of policy would protect against losses due to non-preventable risks, such as those related to weather conditions, pests, or crop diseases, but not from preventable risks related to poor crop management. |
| 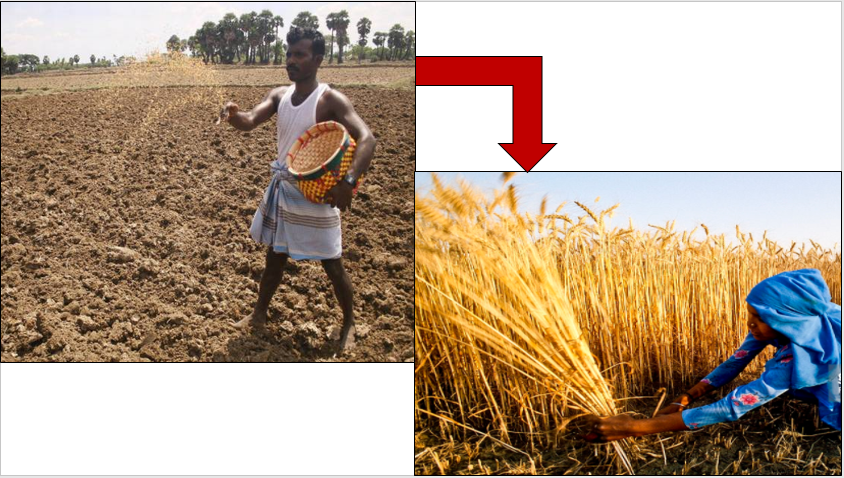 | **Sowing to harvest:** This type of policy pertains to crop losses that are incurred from the time seeds are sown until the crop is harvested, while the crop is standing in the field. This type of policy would protect against losses due to non-preventable risks, such as those related to weather conditions, pests, or crop diseases, but not from preventable risks related to poor crop management. If the policy covers only the sowing to harvest period, it provides no protection for losses that are incurred either before the crop has been sown or transplanted or after the crop has been harvested. |

1. ***Loss Determination:*** This attribute refers to the different modes of loss assessment and three different methods have been included in this experiment, as described below.

| 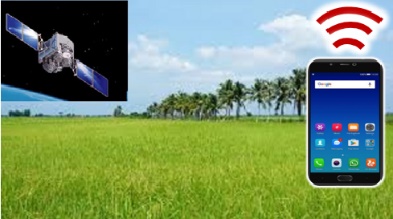 | **Advanced technology:** This method of loss assessment involves the use of technology to assess losses, and could be either through satellite imagery that can detect how green fields are (and hence can assess the health of the underlying crops) or the use of smartphones that can help determine the approximate yield levels at the level of the plot. These advanced technologies can be used to determine losses on a specific farmer’s field, so claims will be specific to a particular farmer. |
| --- | --- |
| 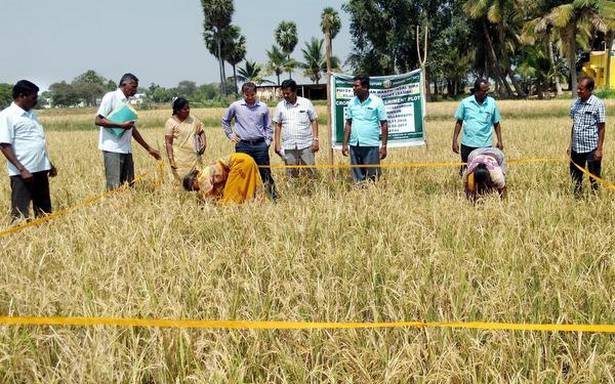 | **Crop-cutting experiment**: This method of loss assessment involves calculating the average yield from a requisite number of sample fields across a village or panchayat and comparing this average yield to a threshold yield. The threshold yield is the average yield over the last 7 years, excluding a maximum of two calamity years. The results of the crop-cutting experiments are used to determine the pay-out eligibility of the represented area in which the crop-cutting exercise is conducted. |
| 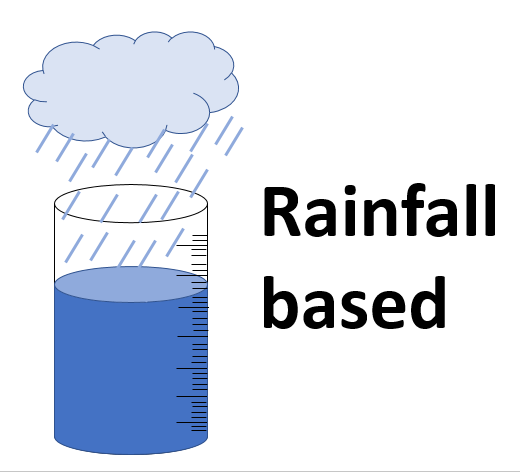 | **Weather-Index based:** This method of loss assessment involves collecting weather data from a centrally-located weather station, and comparing these weather data to some historical threshold. In this exercise, we will assume that an indemnity is decided based on rainfall being less than 75% of the historical average (or greater than 125%) for the time period covered. Results from this method are applicable to all insured persons within the district, irrespective of the actual yields on their plots of land. |

1. ***Timing of Payment:*** This feature refers to the estimated time within which the payments would be made after loss assessment has been conducted. Payouts would be made only if the households are deemed eligible for receiving the payment, irrespective of the loss determination method. Two levels have been considered for this feature, as mentioned below.

| ***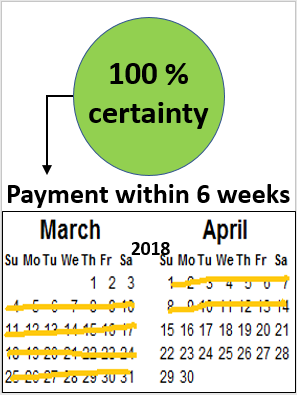*** | **Certain payment within 6 weeks of loss assessment:** This type of policy would provide the insured with the indemnity payment within 6 weeks of the loss assessment, guaranteed. |
| --- | --- |
| ***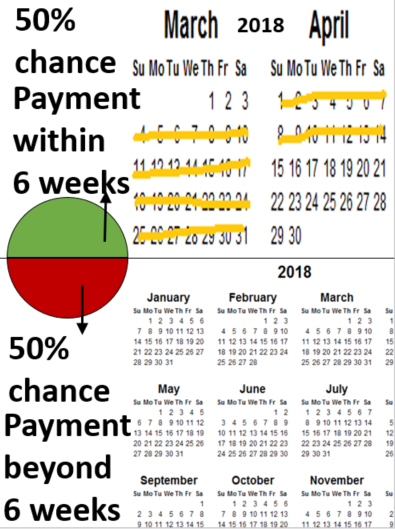*** | **50% chance of payment within 6 weeks, 50% chance payment beyond 6 weeks:** This type of policy is less certain when the claims will be delivered to eligible policyholders. The insurer aims to process claims and issue payments within 6 weeks of assessing a loss, but there is only a 50 percent chance that they will meet this goal. The rest of the time, there will be delays in processing the payment, perhaps up to as long as 6 months, though no comments can be made on when the payments would be made in this case. |

1. ***Insured Sum:*** This feature denotes the maximum amount per acre of land that the insured would be able to receive (conditional on eligibility) after a loss determination is completed. Three different sums insured have been identified considering the average yields and per acre of land for staple crops such as paddy. The sums insured per acre of land that have been included in the study are**: INR 12,000, INR 16,000 and INR 20,000**. These values are the sum insured that would be payable to the farmer in the case of total crop failure, as determined by the method of loss assessment for the insurance policy in question. For any case with yields higher than 0, these values would be proportionately lower.
2. ***Premium Amount:*** Premium amount is determined by actuarial computations that include administrative costs of providing insurance, etc. for this study. The premium is a percentage of the sum insured, but to ease interpretation, the values have been converted into numeric values (sum insured multiplied by the percentage).
